# Intraneural hemangioma of the median nerve: A case report

**DOI:** 10.1186/1749-7221-3-5

**Published:** 2008-02-22

**Authors:** Yunus Doğramacı, Aydıner Kalacı, Teoman Toni Sevinç, Ahmet Nedim Yanat

**Affiliations:** 1 Dept. of Orthopaedics and Traumatology, Mustafa Kemal University Faculty of Medicine, Hatay, Turkey

## Abstract

Hemangiomas of the median nerve are very rare and, so far, only ten cases of intraneural hemangioma of this nerve have been reported in the literature. We present a case of 14-year-old girl who had a soft tissue mass in the region of the left wrist with signs and symptoms of carpal tunnel syndrome. Total removal of the mass was achieved using microsurgical epineural and interfasicular dissection. The symptoms were relieved completely, after this procedure, without any neurologic deficit. On follow-up two years later, no recurrence was observed. Whenever a child or young adult patient presents with CTS the possibility of a hemangioma involving the median nerve should be kept in mind in the differential diagnosis.

## Introduction

The carpal tunnel syndrome (CTS) is the most common neuropathy due to compression seen in adults. There are very few cases in the literature referring to patients of paediatric age [1]. Most of these young patients had a metabolic disorder mucopolysaccharidosis or mucolipidosis. Other unusual causes of CTS in children are fibrolipomas of the median nerve or intraneural perineuroma or haemangioma, haemophilia (secondary to local bleeding), musculotendinous malformation, Klippel-Trenaunay syndrome, Poland's syndrome, scleroderma, benign localised form of gigantism, intensive sports practice, and primary familial CTS [1]. Very rarely Schwannomas of the median nerve can be mistakenly diagnosed and present as carpal tunnel syndrome [2-4]. Lipofibromatous hamartoma of the median nerve at the wrist was reported, and caused macrodactyly of the digits, and also resulted in symptoms of carpal tunnel syndrome [5-7]. Again epithelioid sarcoma of the median nerve may present with symptoms and signs of carpal tunnel syndrome [8]. An isolated malignant peripheral nerve sheath tumor of mild type has also been reported to present with symptoms and signs of carpal tunnel syndrome [9].

Posttraumatic neuroma-in-continuity of the median nerve causing median nerve compression is rare [10]. Damage to the median nerve after vascular graft placement as a result of an occult mass has been documented in a single case [11].

Intraneural hemangioma of the median nerve is a rare condition and only ten cases have been described in the literature [12-20]. Due to mechanical compression, carpal tunnel syndrome (CTS) is the main presenting feature [12-18]. Raynaud's phenomenon may be an associated complaint [16].

Here we present a case of intraneural hemangioma of the median nerve of a 14-year-old female removed surgically by combined interfasicular and epineural resection, no recurrence observed during the two years of postoperative follow-up period.

## Case presentation

A 14-year-old female student presented to our outpatient clinic with painful swelling in the volar surface of the right wrist of 3 years duration; associated with tingling and numbness in the thumb, index, middle and radial half of the ring fingers, difficulty in writing long paragraphs. There was no history of trauma and relevant medical condition.

Physical examination revealed a tender, soft mass, 3 × 5 × 2 cm in dimension in the volar aspect of the right wrist. Tinel sign was positive.

**Figure 1:**
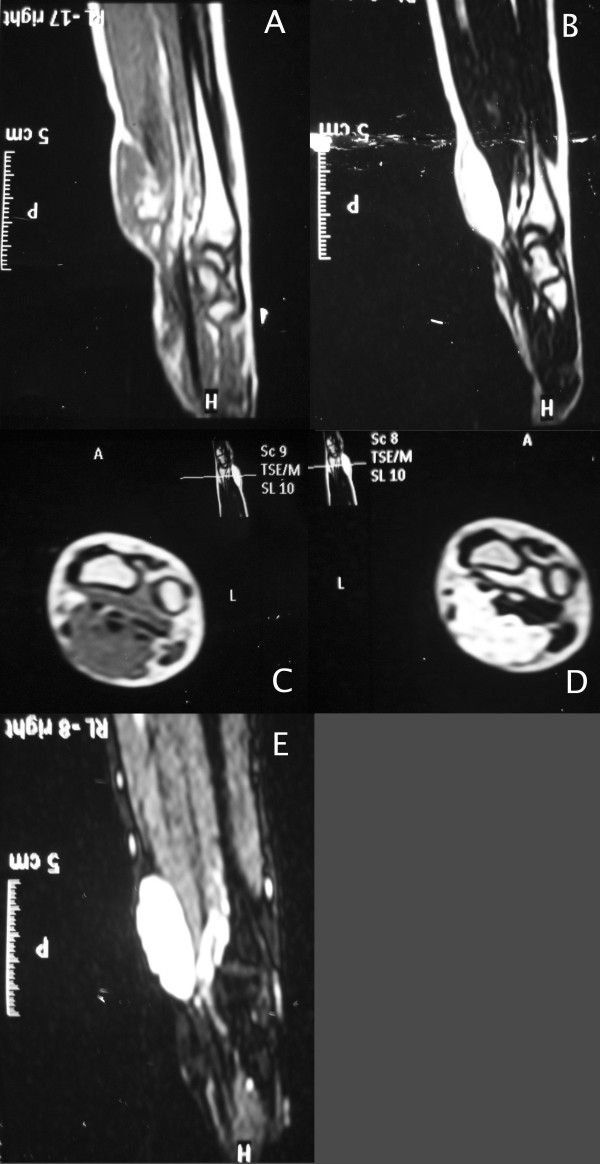
**Magnetic resonance image of intraneural hemangioma.** (A) Sagittal T1 (B) T2 (C) axial T1 and (D) T2 (E) fat suppression images demonstrating an 3 × 2 × 1.5 cm lesion in the volar aspect of the right wrist.

Radiographic examination revealed no bony lesion. Ultrasonographic examination done to exclude any vascular lesion of the radial artery, revealed non pulsatile, cystic mass consistent with ganglion. An MR image obtained in another institution revealed a 3 × 2 × 1.5 cm ovoid rapidly enhancing mass in the volar surface of the right wrist region (Fig. 1).

EMG examination was planned for this patient, but the patient refused to cooperate during the test and the test was not completed successfully.

After preoperative assessment, the patient was admitted for surgical treatment under the diagnosis of volar ganglion causing CTS.

**Figure 2:**
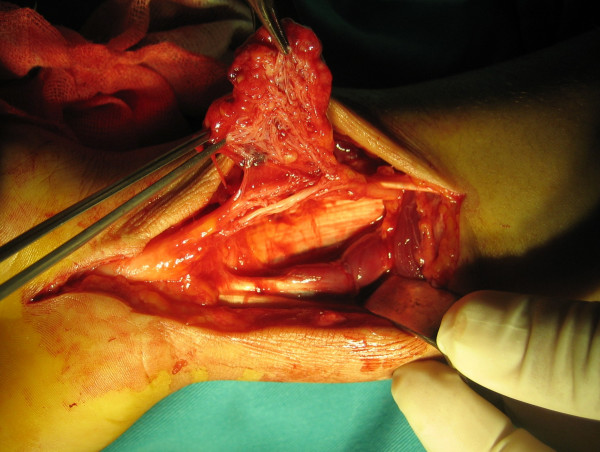
Macroscopic view of the lesion, intraneural and fasicular involvement is obvious.

The operation was done under general anaesthesia, using a pneumatic tourniquet. Exploration revealed a yellowish brown soft tissue mass with areas of hemorrage and dimensions of approximately 4 × 3 × 1.5 cm, originating from the volar surface of the median nerve with intraneural extension and adhesions to the surrounding tissues (Fig. 2). The mass was removed totally by interfasicular and epineural microsurgical resection technique, without structurally damaging the nerve fibbers.

**Figure 3:**
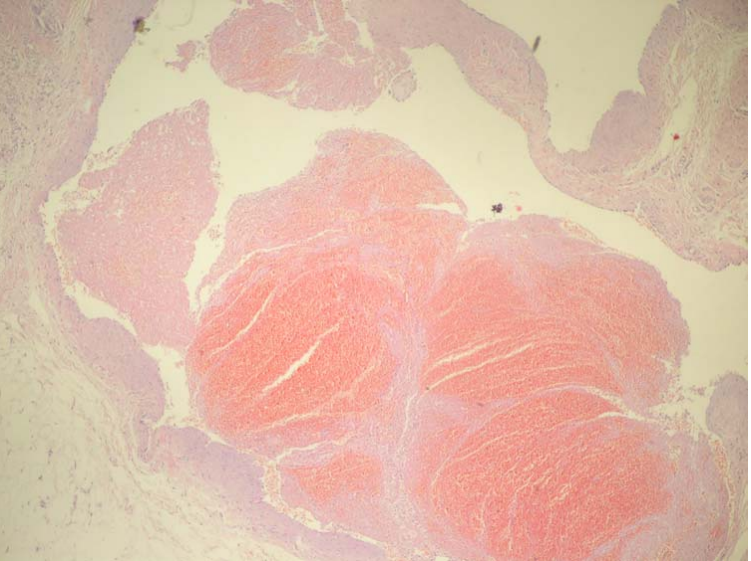
**Microscopic view of hemangioma showing vascular structures in a fibrocollagenous stroma with areas of bleedings.** (Hematoxylin-Eosin, ×100).

Histopathologic and microscopic evaluation revealed dilated and congested vascular structures in a fibrocollagenous stroma with areas of bleedings, consistent with histopathologic findings of hemangioma (Fig 3).

The symptoms were relieved in the first three weeks after the operation. On clinical and ultrasound examination, no recurrence was observed in the first two years following the operation.

## Discussion

Benign intraneural hemangioma originating from peripheral nerves is rare. Most patients present in with a painful, soft mass along the path of a nerve with signs and symptoms of nerve compression and entrapment.

A thorough search through the literature revealed ten cases of hemangioma of the median nerve [12-19]. In all the described cases CTS is the presenting feature and in one case Raynaud's phenomenon was an associated presenting feature.

The tumor may not be easily recognised until it becomes painful and it is rarely diagnosed before surgery. In the differential diagnosis, lipoma, lipofibroma, hamartoma and intraneuronal Schwannoma must be considered [20,21].

Ultrasonography may give useful information about the nerve's dynamic relation to the surrounding musculotendinous structures [22] and nerve conduction studies may reveal non specific features of compressive neuropathies [23]. For appropriate planning of surgical therapy and preoperative diagnosis, MRI is essential and gives useful information regarding tumor location, size, extent and relationship of peripheral nerve.

Hemangioma shows a hyperintense signal on T1- and T2- weighted images with fat suppression sequences. Flow voids are usually apparent and feeding vessels may be visualized; these lesions are also noted to enhance after Gd-addition. On angiography an early and persistent tumoral blush is demonstrated [20].

Schwannoma is a slightly hypodense, solid tumor with no vascular contrast enhancement on CT. MRI shows intermediate signals on. T1-W, and T2-W imaging shows high signal intensity with some heterogenity [24]. Lipomas exhibit signal characteristics consistent with those of normal adipose tissue: homogeneous hyperintensity on T1- and T2-weighted sequences [25]. MR imaging findings of lipofibromatous hamartoma are pathognomonic which consist of serpiginous T1- and T2-weighted low-intensity structures containing and surrounded by fat (hyperintense on T1- and hypointense on T2-weighted fat suppression sequences), giving the lesion a spaghetti-like appearance on sagittal images, and a "coaxial cable-like" appearance on coronal images [26].

No certain protocol has been established to manage this difficult condition, however conservative treatment usually fails and surgery is the treatment of choice. When possible total resection of intraneural hemangiomas is curative, partial resection may relieve symptoms but recurrence may occur which may require en-bloc nerve resection and repair with nerve graft [14].

The longest period of follow-up without recurrence has been reported by Oztekin et al. [18]. They reported a case of CTS due to a cavernous hemangioma of the median nerve, which was successfully removed by epineural resection, and no recurrence was observed over a 6 year follow-up period. Patel et al. [14] reported two cases of hemangioma of the median nerve which they treated by partial excision and resulted in recurrence in the third year, one of the recurred case managed by resection of median nerve and nerve grafting without recurrence, four years after surgery.

Chatillon et al. [20] reported the first case of using radiotherapy in the treatment of intraneural hemangioma. Preoperative embolization and postoperative radiotherapy combined with partial resection were beneficial in a case of intraneural hemangioma involving inferior trunk of brachial plexus and resulted in symptomatic relief and radiologic shrinkage in the size of the mass seen on serial follow-up MRI images, with a follow-up period of two years.

In our case, total resection of the hemangioma was achieved by combined epineural resection and interfasicular dissection with microsurgical resection technique, no neurologic complications observed postoperatively and no recurrence observed in the two year follow-up period.

The type of microsurgical dissection and resection should be decided at the time of surgery and careful preoperative planning using MRI, and if needed angiography, is essential for cystic lesions of the volar side of wrist. Excision of the affected nerve and grafting should be the last choice and should only be used in complicated cases and when there are frequent recurrences.

## Conclusion

Whenever a child or young adult patient presents with CTS the possibility of a hemangioma involving the median nerve should be kept in mind in the differential diagnosis.
